# Supplementary material for: Sex-dependent differences in the genomic profile of lingual sensory neurons in naïve and tongue-tumor bearing mice
Source: Sci Rep. 2023 Aug 12;13:13117. doi: 10.1038/s41598-023-40380-6 (PMC10423281; doi:10.1038/s41598-023-40380-6)
Supplement: Supplementary file 1 — Supplementary Information. [file 41598_2023_40380_MOESM1_ESM.docx]

| **Sample** | **Sorted Cells Collected (Mean± SEM)** | **RIN value of RNA**  **(Mean± SEM)** |
| --- | --- | --- |
| Male Normal(MN) | 10333± 2667 | 7.567± 0.088 |
| Male Tumor (MT) | 12667± 2963 | 7.267± 0.49 |
| Female Normal (FN) | 23333± 3383 | 7.233± 0.466 |
| Female Tumor-1 (FT1) | 20667± 5364 | 7.100± 0.23 |

**Supplementary Table 1.** Number of sorted cells and RIN values of RNA of all samples within four groups. Data represented as mean ± SEM.MN: Male Normal. MT: Male Tumor. FN: Female Normal, FT: Female Tumor. N=3 per group.

| **Gene** | **Description** | **MN**  **(RPKM)** | **FN**  **(RPKM)** | **FC (FN vs MN)** | **P-value** |
| --- | --- | --- | --- | --- | --- |
| **Upregulated** | | | | | |
| Tsix | X (inactive)-specific transcript, opposite strand | 0.011 | 8.547 | 777 | 0.014 |
| Sprr1a | small proline-rich protein 1A | 3.15 | 39.293 | 12.474 | 0.018 |
| C1qa | complement component 1, q subcomponent, alpha polypeptide | 2.318 | 28.027 | 12.091 | 0.023 |
| Csf1r | colony stimulating factor 1 receptor | 1.03 | 12.28 | 11.922 | 0.014 |
| C1qc | complement component 1, q subcomponent, C chain | 2.935 | 25.993 | 8.856 | 0.0014 |
| S100a9 | S100 calcium binding protein A9 (calgranulin B) | 5.096 | 18.113 | 3.554 | 0.005 |
| Retnlg | resistin like gamma | 1.862 | 6.106 | 3.279 | 0.012 |
| Dnahc1 | dynein, axonemal, heavy chain 1 | 4.994 | 15.93 | 3.19 | 0.004 |
| D**ownregulated** | | | | | |
| Fam23a | transmembrane protein 236 | 6.654 | 0.685 | 0.103 | 0.025 |
| Ddx3y | DEAD box helicase 3, Y-linked | 12.885 | 0.03 | 0.002 | 0.028 |

**Supplementary Table 2.** Top 10 DEGs of FN vs MN.

| **Gene** | **Description** | | **MT**  **(RPKM)** | **MN**  **(RPKM)** | **FT**  **(RPKM)** | **FN**  **(RPKM)** | **FC**  **( MT vs MN)** | **FC**  **(FT vs FN)** | **P value**  **(MT vs MN)** | **P value**  **( FT vs FN)** |
| --- | --- | --- | --- | --- | --- | --- | --- | --- | --- | --- |
| **Genes Upregulated in MT vs MN and FT vs FN** | | | | | | | | | | |
| Sprr1a | small proline-rich protein 1A | | 60.133 | 3.15 | 93.055 | 39.293 | 19.09 | 2.368 | 0.00 | 0.00 |
| Gal | galanin and GMAP prepropeptide | | 402.83 | 78.825 | 697.683 | 349.2 | 5.11 | 1.998 | 0.00 | 0.02 |
| Chi3l3 | chitinase-like 3 | | 30.434 | 10.121 | 45.54 | 12.853 | 3.007 | 3.543 | 0.01 | 0.00 |
| Cxcl10 | chemokine (C-X-C motif) ligand 10 | | 10.051 | 4.189 | 6.454 | 2.582 | 2.399 | 2.5 | 0.04 | 0.01 |
| Apod | apolipoprotein D | | 93.495 | 39.85 | 315.912 | 120.196 | 2.346 | 2.628 | 0.00 | 0.00 |
| Timp1 | tissue inhibitor of metalloproteinase 1 | | 25.59 | 11.59 | 50.654 | 26.675 | 2.208 | 1.899 | 0.00 | 0.02 |
| Tnfsf9 | tumor necrosis factor (ligand) superfamily, member 9 | | 5.612 | 2.692 | 10.624 | 4.364 | 2.085 | 2.434 | 0.04 | 0.01 |
| Mustn1 | musculoskeletal, embryonic nuclear protein 1 | | 8.656 | 4.671 | 14.756 | 8.275 | 1.853 | 1.783 | 0.00 | 0.04 |
| Mlf1 | myeloid leukemia factor 1 | | 7.528 | 4.19 | 8.119 | 4.21 | 1.797 | 1.929 | 0.01 | 0.04 |
| Gadd45b | growth arrest and DNA-damage-inducible 45 beta | | 22.097 | 12.905 | 32.249 | 18.622 | 1.712 | 1.732 | 0.00 | 0.04 |
| **Genes Upregulated in MT vs MN and downregulated FT vs FN** | | | | | | | | | | |
| Fam107a | family with sequence similarity 107, member A | | 6.914 | 3.697 | 3.615 | 8.328 | 1.87 | 0.434 | 0.03 | 0.03 |
| Sgk1 | serum/glucocorticoid regulated kinase 1 | | 26.42 | 17.516 | 12.38 | 28.117 | 1.508 | 0.44 | 0.00 | 0.04 |
| Ctgf | cellular communication network factor 2 | | 57.387 | 29.272 | 20.413 | 74.014 | 1.96 | 0.276 | 0.04 | 0.00 |
| **Genes Downregulated in MT vs MN and Upregulated FT vs FN** | | | | | | | | | | |
| Scgb1a1 | | secretoglobin, family 1A, member 1 (uteroglobin) | 3.968 | 8.572 | 24.357 | 11.736 | 0.463 | 2.075 | 0.02 | 0.02 |
| Fabp7 | | fatty acid binding protein 7, brain | 154.989 | 322.27 | 847.997 | 391.936 | 0.481 | 2.164 | 0.00 | 0.02 |

**Supplementary Table 3.** DEGs common to MT vs MN and FT vs FN

| **Biological Process** | **DEGs** |
| --- | --- |
| Second Messenger Signaling | Gal, Mt1, Avpr1a, Irgm1, Igtp, Mt2 |
| IL1-Signaling | Irak3, Rps6ka5, Egr1 |
| Pattern Recognition Signaling | Tifa, Irgm1, Irak3, Igtp |
| Response to Interferon-Gamma | Irgm1, Igtp |
| Inflammatory Response | Fas, Timp1, Csf1, chi3l3,Gal, Cxcl10, Serpinb1a,Rps6ka5,Anxa1 |
| Immune Process | Fas, Fst, Tifa, Myc, csf1, Zfp36, Rps19, Nts, cxcl10, Gbp, Fas, Mif1, Irgm1, Igtp, Dll4, tnfsf9,Cebpd,Fos, Hes, Egr1,Anxa1, |
| Leucocyte Differentiation | Fasn, Csf1, Fas, Dll4, tnfsf9, Fos, Egr1, Anxa1 |
| Leucocyte Migration | Csf1, Rps19, cxcl10, Anxa1 |
| Regulation of Ion Transport | Gem, Fgf23, Usp2, Cckar, Gal, cxcl10, Avpr1a, Sgk1, Per1, Hes1 |
| Autophagosome Assembly | Irgm1 |
| Glial Cell Proliferation | Igtp |
| Response to Glucocorticoid Stimulus | Zfp36, Fam107a, Fas, Sgk1, Anxa1 |
| Circadian Regulation | Fas, gm129, Per1, Bhlhe40, Egr1 |
| Apoptosis | Timp1, Myc, Phlda1, Zfp36, Mt1, Cxcl10, Ier3, fas, Socs3, Sgk1, Irak3, Gadd45b, Egr1, Anxa1,Spink2, hspa1a |

**Supplementary Table 4.** Biological Processes associated with DEGs upregulated in MT vs MN**.**

| **Biological Process** | **DEGs** |
| --- | --- |
| Chemokine Signaling Pathway | Cxcl2, Cxcl12, Ccl12, ccl11, Pf4, cxcl1, Cxcl10, Ccl7, Ccl4, Ccl3, |
| MAPK Signaling | Gadd45a, IL1b, Sfpi1, Gadd45b |
| Lipopolysaccharide Signaling Pathway | Ccl12, Ly86, IL1b, cd14, Sfpi |
| Interferon Gamma Signaling | Ccl12, cd74, Ccl11, Ccl7, ifitm1, H2-Eb1, ccl4, Rab20, h2Ab1, H2-Aa, Ccl3 |
| IL-1 Signaling | Ccl12, Rbmxrt, Il1b, Ccl11, Ccl7, Ccl4, Ccl3 |
| Immune Process | Cd83, Gm8909, C1qc, ccl12, cd74, casp6, Kcnn4, Fcgr2b, Pgf, Il1b, Icam1, cxcl1, Scgb1a1,Gal,Cacl10,Hmox1,cd14, ccl7, npy,Sfpi1, BC013712, Apod,Fcer1g, H2-Eb1,tnfsf9,H2-Q6, Lgals1, H2-Ab1, H2-Aa, Ccl3 cmtm3 |
| Humoral Immune Response | Cxcl2. ccl12, Pf4, Cxcl1, Cxcl10, Npy, camp,S100a9, |
| Leucocyte Migration | Cxcl2, xxl12, Il1b, Icam1, ccl11, Pf4, Spp1, cxcl1, cxcl10,ccl7, Fcer1g, ch25h,ccl4, S100a8,S100a9,ccl3 |
| Antigen Processing and Presentation | Gm8909,cf74, Fcgr2b, Icam1, Fcer1g,H2-Eb1,H2-Q6, H2-Ab1, H2-Aa, Ctss |
| Inflammatory Response | Timp1, cxcl2, ccl12, Chi3l1, Ly86, Casp6, Il1b,chi3l3,Icam1, ccl11, pf4, cacl1,gal, cxcl10, Hmox1,ccl7, BC013712, CampFcer1g, Ccl4,S100a8,S10a9, Ccl3 |
| Regulation of Cytokine Production | Cd83, chi3l1, cd74, IL1b, chi3l3, Lum, cd14, camp, Fcer1g, Tnfsf9, ccl4,ccl3 |
| RNA Splicing | Rnf113a2, rnf113a1, Exosc5 |
| Apoptosis | Gadd45a, Txndc12, timp1, Ccl12, Cd74, Casp6, Fcgr2b, Glrx, IL1b, Icam1, Spp1, Gal,Rps7, Cxcl10, Hmox1,Nme5, Bag1, Npy, Cryab, Gadd45b, Lgals1,Hspb6,S100a8,Atp5g1,S100a9,Ccl3,Plekhf1 |
| Cell Activation | Cd83, timp1, cd74, Fcgr2b, IL1b, Icam1, Pf4,Scgb1a1, Ptgds, Cxcl10,Npy,Sfpi1, Camp,Pcer1g,Tnfsf9, Lgals1, H2-Ab1 |
| Transport Regulation | Ccl12, cf74, Casp6, Kcnn4, Fcgr2b, Glrx, IL1b, Icam1, Rab13, Spp1, cxcl1,gal,cxcl10, Hmox1, cd14,Car7, Sfpi1,Apod, Aqp2,Cryab, Dcn,Fcer1g,S100a8,Vtn,Fxyd3, Apoc1,Ctss |
| Positive Regulation of Angiogenesis | Gadd45a, Chi3l1, Dcn, Ngp, Pgf, IL1b, Ccl11, cxcl10,Hmox1, Camp,Hspb6 |

**Supplementary Table 5.** Biological Processes associated with DEGs upregulated in FT vs FN.

| **Biological Process** | **DEGs** |
| --- | --- |
| Negative Regulation of Angiogenesis | Tspan12, tek, Ctgf, Col18a1, Robo4, Mmrn2, Calcrl,Lef1,Pecam1 |
| Cytoskeleton Organization | Fli1, Gmfb, Tek, Ppm1f, Spry4, Ctgf, Fam107a, Sgk1,Cdh5, Arfip1, Scin, Pecam1 |
| Locomotion | Tek, Ppm1f, Sema3g, Col18a1, Ptprm, Fam107a, Nedd9, Robo4,mmrn2, F2r, Cdh5, Net1,Arrdc3, Mctp1,Patz1,Lef1,Pecam1 |
| Cell Adhesion | Tek, Thsd1,Bmx, Ctgf, Col18a1, Ptprm,Nedd9, Tmem47, Robo4, Mmrn2, Cdh5, net1,Lef1, pecam1, |

**Supplementary Table 6.** Biological Processes associated with DEGs downregulated in FT vs FN


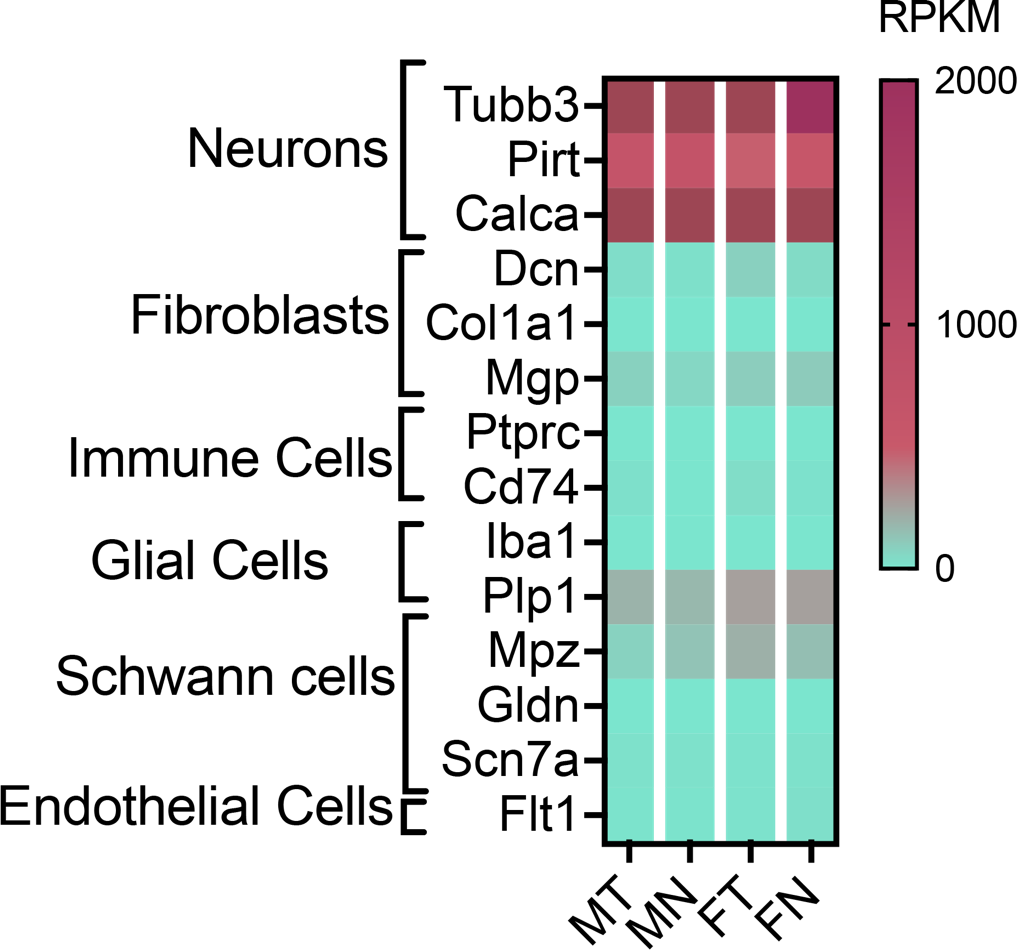


**Supplementary Figure 1. Confirmation of neuronal enrichment of samples following FACS sorting.** Bulk-RNA sequencing was performed from FACS sorted retrolabeled lingual trigeminal neurons. Heatmap of RPKM values in MT, MN, FT and FN, for specific neuronal and non-neuronal markers such as fibroblasts, immune cells, glial cells, Schwann cells and endothelial cells is shown.


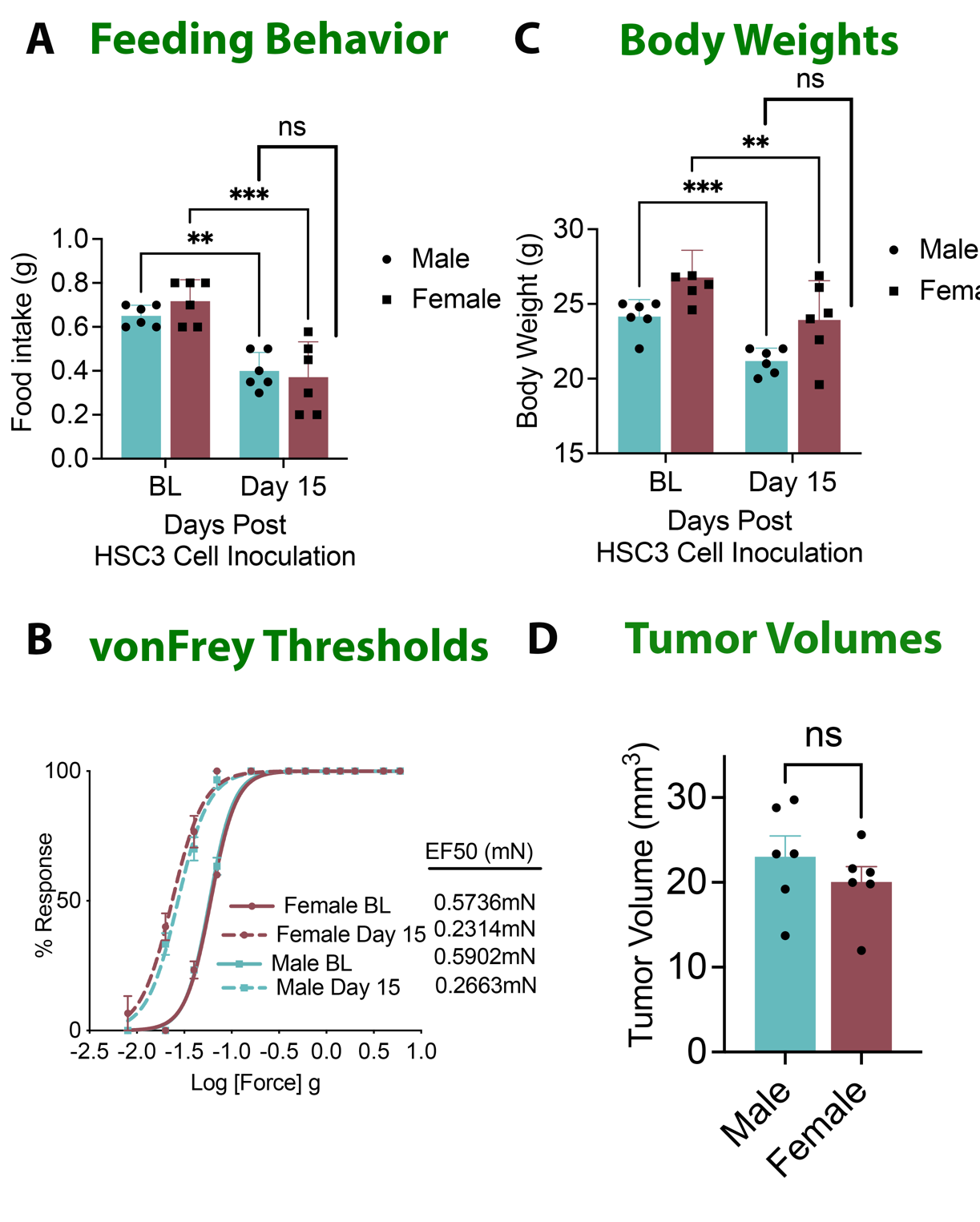


**Supplementary Figure 2. Pain Behavior post-HSC3 Tongue Tumor Growth in Males and Females.** Male and Female mice were injected with 3.5x10^5 HSC3 cells in the tongue. **A**. Feeding Behavior and **B.** von Frey Thresholds in the vibrissal pad were determined at baseline (BL) and at day 15 post cell-inoculation. N=6. Data are presented as mean ± SEM and analyzed by one-way ANOVA with Sidak’s post-hoc test p<0.05 for feeding behavior and as stimulus response curves for von Frey thresholds with calculated EF50 values. **C.** Body weights were determined at baseline (BL) and at day 15 post cell-inoculation. N=6. Data are presented as mean ± SEM and analyzed by one-way ANOVA with Sidak’s post-hoc test p<0.05. **D**. Tumor Volumes were measured at day 15 post cell-inoculation. N=6. Data are presented as mean ± SEM and analyzed by Unpaired Student’s T-test at p<0.05.
